# Composition, Development, and Function of Uterine Innate Lymphoid Cells

**DOI:** 10.4049/jimmunol.1500689

**Published:** 2015-09-14

**Authors:** Jean-Marc Doisne, Elisa Balmas, Selma Boulenouar, Louise M. Gaynor, Jens Kieckbusch, Lucy Gardner, Delia A. Hawkes, Cynthia F. Barbara, Andrew M. Sharkey, Hugh J. M. Brady, Jan J. Brosens, Ashley Moffett, Francesco Colucci

**Affiliations:** * Department of Obstetrics and Gynecology, University of Cambridge School of Clinical Medicine, National Institute for Health Research Cambridge Biomedical Research Centre, Cambridge CB2 0SW, United Kingdom;; † Centre for Trophoblast Research, University of Cambridge, Cambridge CB2 1QP, United Kingdom;; ‡ Department of Pathology, University of Cambridge, Cambridge CB2 1QP, United Kingdom;; § Department of Life Sciences, Imperial College London, London SW7 2AZ, United Kingdom; and; ¶ Division of Reproductive Health, University of Warwick, Coventry CV2 2DX, United Kingdom

## Abstract

Innate lymphoid cells (ILCs), including NK cells, contribute to barrier immunity and tissue homeostasis. In addition to the role of uterine NK cells in placentation and fetal growth, other uterine ILCs (uILCs) are likely to play roles in uterine physiology and pathology. In this article, we report on the composition of uILCs in the endometrium during the luteal phase and in the decidua during early pregnancy. Whereas nonkiller uILC1s and uILC2s are barely detectable in mouse and not detected in humans, a sizeable population of uILC3s is found in human endometrium and decidua, which are mostly NCR^+^ and partially overlap with previously described IL-22–producing uterine NK cells. Development of mouse uILC3 is Nfil3 independent, suggesting unique features of uILCs. Indeed, although the cytokine production profile of mouse uILCs recapitulates that described in other tissues, IL-5, IL-17, and IL-22 are constitutively produced by uILC2s and uILC3s. This study lays the foundation to understand how ILCs function in the specialized uterine mucosa, both in tissue homeostasis and barrier immunity and during pregnancy.

## Introduction

Innate lymphoid cells (ILCs) express transcription factors and produce cytokines mirroring those of adaptive T cell subsets (1–3). Group 1 ILCs (ILC1) include IFN-γ–producing cells that express T-bet, similarly to conventional NK cells (cNK). Unlike cNK cells, nonkiller ILC1s do not express Eomes. Eomes^-ve^ ILC1s can be found in the spleen, liver (4), and intestine (5). Group 2 ILCs (ILC2) include CD127 (IL-7Rα) and GATA-3–expressing cells previously known as nuocytes, innate helper 2 cells, or natural helper cells (6). ILC2s express ST2 (IL-33R) and IL-17RB (IL-25R) and produce IL-5 and IL-13, but also amphiregulin, IL-9, IL-10, IL-6, and little IL-4. They are found in fat-associated lymphoid clusters, lymph nodes, intestine, and airways and are involved in lung inflammation, airways hyperreactivity, and asthma. ILC2s also participate in clearance of intestinal parasitic worms and in wound healing after influenza infection. Group 3 ILCs (ILC3) express RORγt and produce IL-17A and/or IL-22 (7). This group is composed of three main classes in mice. One is CCR6^+^ lymphoid tissue inducers (LTis), which orchestrate lymphoid organogenesis. CCR6^−^NCR^+^ ILC3s are found in the gut and produce IL-22, but not IL-17A, playing a major role in gut immunity and mucosal homeostasis. Upon stimulation with IL-12 and IL-18, NCR^+^ ILC3s can downregulate RORγt, upregulate T-bet, and differentiate into ILC3-derived ILC1s (8–10). CCR6^−^NCR^−^ ILC3s have been also described that can expand in the inflamed colon and produce IL-17A, IL-22, and IFN-γ (11).

The basic leucine zipper transcription factor Nfil3/E4BP4 was reported as master regulator of NK cell differentiation (12, 13) and was subsequently found to have a broader role in the immune system (14). For example, Nfil3 is required for the development of ILCs (15–17). *Nfil3^−/−^* mice exhibit defects in intestine, spleen, and liver ILC1s, in lung, intestine, and adipose tissue ILC2s, as well as in intestine and spleen ILC3s. Peyer’s patches (PP) and cryptopatches are abnormal and not fully developed in *Nfil3^−/−^* mice, whereas lymph nodes appear normal (15–18). Interestingly, tissue-resident NK (trNK) cells like liver, salivary glands, skin, and uterus, as well as thymically derived NK cells, are present in *Nfil3^−/−^* mice (19–21). Therefore, not all ILCs are dependent on Nfil3 for development.

Immune cells are important for reproduction. NK cells, dendritic cells, macrophages, and regulatory T cells mediate key processes (22), but other lymphoid cells may also contribute, including ILCs (23). The human endometrium undergoes tissue remodeling in each menstrual cycle and even more dramatically when the mucosa decidualizes during pregnancy. Innate immune cells, including uterine NK (uNK) cells and macrophages, are dominant in the decidua during pregnancy in both mice and humans (24) and probably contribute to tissue homeostasis and preparation for implantation. The decidua basalis is the site where fetal placental trophoblast cells invade and interact with uNK cells. The probable role of uNK cells is to remodel the uterine arteries during pregnancy (22, 25). Lymphoid aggregates (LA) mostly composed of noncytotoxic CD8^+^ T cells form regularly in the basal layer of the endometrium during the menstrual cycle in humans, although the origin and function of LA is unclear (26).

In the virgin female mouse, the uterus is the most NK-rich organ in the body with uNK cells representing up to 50% of lymphocytes. During pregnancy, uNK cells continue to be a major population and increase in absolute numbers, although less represented (20–30%). The role of uNK cells in mouse uterine vascular remodeling and fetal-placental growth is well established (27). The decidua proximal to the invading trophoblast is rich in uNK cells. Distal to the trophoblast and in between the two smooth muscle layers of myometrium (Myo) is the mesometrial LA of pregnancy (MLAp), which is also rich in uNK cells. It is unclear how the MLAp forms and develops and what its function may be (28).

A recent report described ILCs in human decidua (23). We analyzed which ILCs are present in the nonpregnant human and mouse endometrium and whether they change in composition and function during pregnancy. We also studied the developmental requirement of mouse uterine ILCs (uILCs), revealing uterine-specific, Nfil3-independent ILC3s.

## Materials and Methods

### Mice

C57BL/6 mice were purchased from Charles River. *Rag2^−/−^* and *Nfil3^−/−^* mice were previously described (12, 29). Mice were used at 8–12 wk old for tissue analysis or for time matings. All mice were bred at the University of Cambridge Central Biomedical Service under pathogen-free conditions and housed according to U.K. Home Office guidelines.

### Human samples

Decidual biopsies were obtained from donors undergoing elective termination between 7 and 12 wk of pregnancy. The Cambridge Research Ethics Committee approved this study (04/Q0108/23 and 08/H0305/40). Endometrial biopsies were timed between 6 and 10 d after the preovulatory luteinizing hormone surge. All endometrial biopsies were obtained in ovulatory cycles, and none of the subjects was on hormonal treatments for at least 3 mo before the procedure. Subjects were recruited from the Implantation Clinic, a dedicated research clinic at University Hospitals Coventry and Warwickshire National Health Service Trust. The study was approved by the National Health Service National Research Ethics–Hammersmith and Queen Charlotte’s & Chelsea Research Ethics Committee (1997/5065). Written, informed consent was obtained from all participants in accordance with the guidelines in the Declaration of Helsinki (2000).

### Tissue processing

Mouse uterus, spleen, liver, lung, PP, and lymph nodes were processed using enzymatic protocol. Minced tissues were incubated 2 × 15 min with HBSS (PAA), 10% FCS (Life Technologies), 5 mM EDTA (Sigma), 15 mM HEPES solution (Life Technologies) on a rotator at 37°C, then digested during 30 min with RPMI 1640 containing 2% FCS, 30 μg/ml DNase I (Roche), and 0.1 WU/ml Liberase DH (Roche). Digested tissues were filtered and smashed with a syringe plunger on a cell strainer to mechanically dissociate the remaining bits of tissues. Leukocytes were enriched on an 80%/40% Percoll (GE Healthcare Life Sciences) gradient. Human decidua and endometrium tissues were mechanically processed. Leukocytes were enriched by layering on Lymphoprep (Axis-Shield).

### Flow cytometry

Conjugated Abs anti-mouse CD45 (clone 30-F11), CD3ε (500A2 or 17A2), CD19 (6D5), CD11b (M1/70), NK1.1 (PK136), NKp46 (29A1.4), CD90.2 (30-H12), ST2 (DIH9 and RMST2-2), ICOS (C398.4A), CD25 (PC61), c-kit (2B8), Sca-1 (D7), IL-17RB (752101), CCR6 (29-2L17), IFN-γ (XMG1.2), IL-5 (TRFK5), IL-13 (eBio13A), IL-22 (Poly5164), IL-17A (TC11-18H10.1), RORγt (Q31-378), Eomes (Dan11mag), and CD16/32-Fc blocking (93); Abs anti-human CD45 (HI30), CD3 (UCHT1), CD19 (HIB19), CD14 (M5E2), CD127 (A019D5), CD9 (M-L13), CD56 (HCD56), CRTH2 (BM16), c-kit (104D2), NKp44 (P44-8), RORγt (Q21-559) and human Fc blocking; anti-human/mouse GATA-3 (TWAJ and 16E10A23) and T-bet (O4-46) were purchased from Biolegend, eBioscience, BD Biosciences, or R&D Systems. Cytokines and transcription factors were stained using the Foxp3 staining buffer set (eBioscience) according to the manufacturer’s instructions. Fixable viability dyes eFluor 780 and eFluor 506 (eBioscience) were used to exclude dead cells. Samples were acquired on an LSR Fortessa (BD Biosciences) using FACSDiva and analyzed using FlowJo (Tree Star).

### Cytokine activation and intracellular staining

Isolated leukocytes were activated with 50 ng/ml PMA (Sigma) and 500 ng/ml Ionomycin (Sigma) during 4 h or with the following cytokines at the indicated concentrations and incubation times at 37°C: 10 ng/ml recombinant murine IL (rmIL)-12 and 50 ng/ml rmIL-15, 50 ng/ml rmIL-1β and 50 ng/ml rmIL-23 during 4 h in the presence of brefeldin A (eBioscience) and monensin (Sigma); 20 ng/ml rmIL-25 and 20 ng/ml rmIL-33 during 18 h, brefeldin A and monensin were added during the last 6 h. All cytokines were purchased from Peprotech except rmIL-23 obtained from R&D Systems. Then cells were stained for cell-surface markers, intracellular cytokines, and transcription factors.

### Immunohistochemistry

Sections of cryoembedded uterine tissue from pregnant females at gestation day (gd) 9.5 were cut at 7 μm and fixed in acetone. Sections were blocked with normal goat serum, followed by avidin and biotin blocking solutions to minimize nonspecific binding. Sections were incubated with anti-mouse CCR6-PE (29-2L17), CD127–Alexa Fluor 647 (A7R34), and CD3-biotin (145-2C11) or isotype controls for 2 h at room temperature, and for a further 30 min with streptavidin conjugated to Alexa Fluor 488 (Life Technologies). Sections were counterstained with DAPI. All Abs were purchased from Biolegend except where indicated. Images were acquired using a Leica SP5 confocal microscope and analyzed using ImageJ software to adjust brightness and contrast.

### Statistics

Two-tailed, unpaired or paired Student *t* test (GraphPad Prism 6) was used to statistically analyze the data: *p ≤ 0.05, **p < 0.01, ***p < 0.001, ****p < 0.0001. All data are expressed as means ± SEM.

## Results

### uILC3s in the human uterine mucosa

**FIGURE 1.**
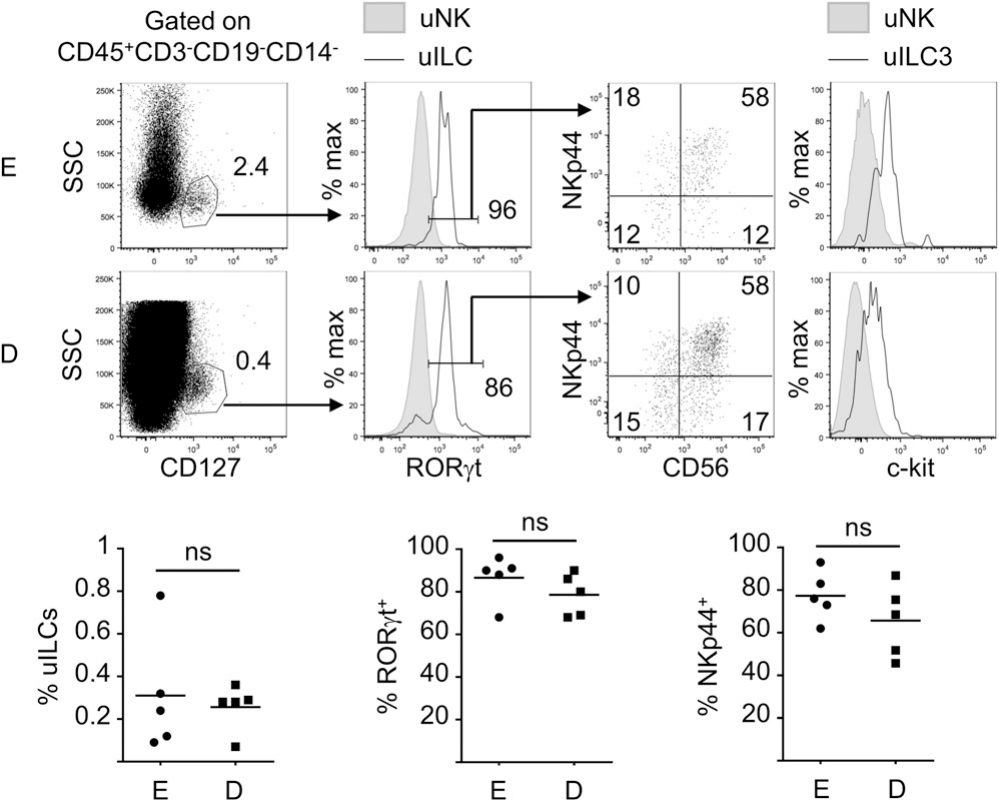
uILC3 is the major subset in human endometrium and decidua. *Top panels* show representative data from human endometrium (E) and decidua (D). CD127^+^ uILCs were gated on CD45^+^CD3^−^CD19^−^CD14^−^ cells and analyzed for intracellular RORγt expression. RORγt^+^ uILC3s were assessed for NKp44, CD56, and c-kit expression. *Bottom panels* show percentages of CD127^+^ uILCs among leukocytes (*bottom left*), RORγt^+^ uILC3s among CD127^+^ cells (*bottom middle*), and NKp44^+^ among RORγt^+^ uILC3s (*bottom right*) in the E and the D (*n* = 5 samples for each group, mean, unpaired *t* test).

We analyzed samples obtained from first-trimester decidua and from endometrium of nonpregnant women during the luteal phase. Tissues mechanically dissociated as enzymatic digestion resulted in loss of CD56 and NKp44 expression (data not shown). Human uNK cells have been extensively characterized and, therefore, were not analyzed in this study. CD3^−^CD19^−^CD14^−^CD127^+^ cells were found in both endometrium and decidua and with similar frequencies (Fig. 1). The vast majority of these cells expressed RORγt, and thus belong to uILC3s (Fig. 1). NCR^+^ uILC3s was the major subset, expressing also higher levels of CD56 compared with NCR^−^ uILC3s (Fig. 1). Unlike mature uNK cells, uILC3s were found to be c-kit^+^ and CD9^−^. CD127^+^ uILC2s (CD56^−^RORγt^−^GATA-3^hi^CRTH2^+^) were detectable in three of five samples at a very low frequency (<0.01%; data not shown). No CD127^+^ uILC1s (RORγt^−^T-bet^+^) could be detected in these samples (data not shown). These results demonstrate the presence of a dominant population of NCR^+^CD127^+^ uILC3s, with a few NCR^−^ cells. These data confirm a recent study analyzing ILCs in the decidua (23) and show for the first time, to our knowledge, that the uILC composition is similar in nonpregnant endometrium and decidua.

### Development of mouse uILC1s does not depend on Nfil3

**FIGURE 2.**
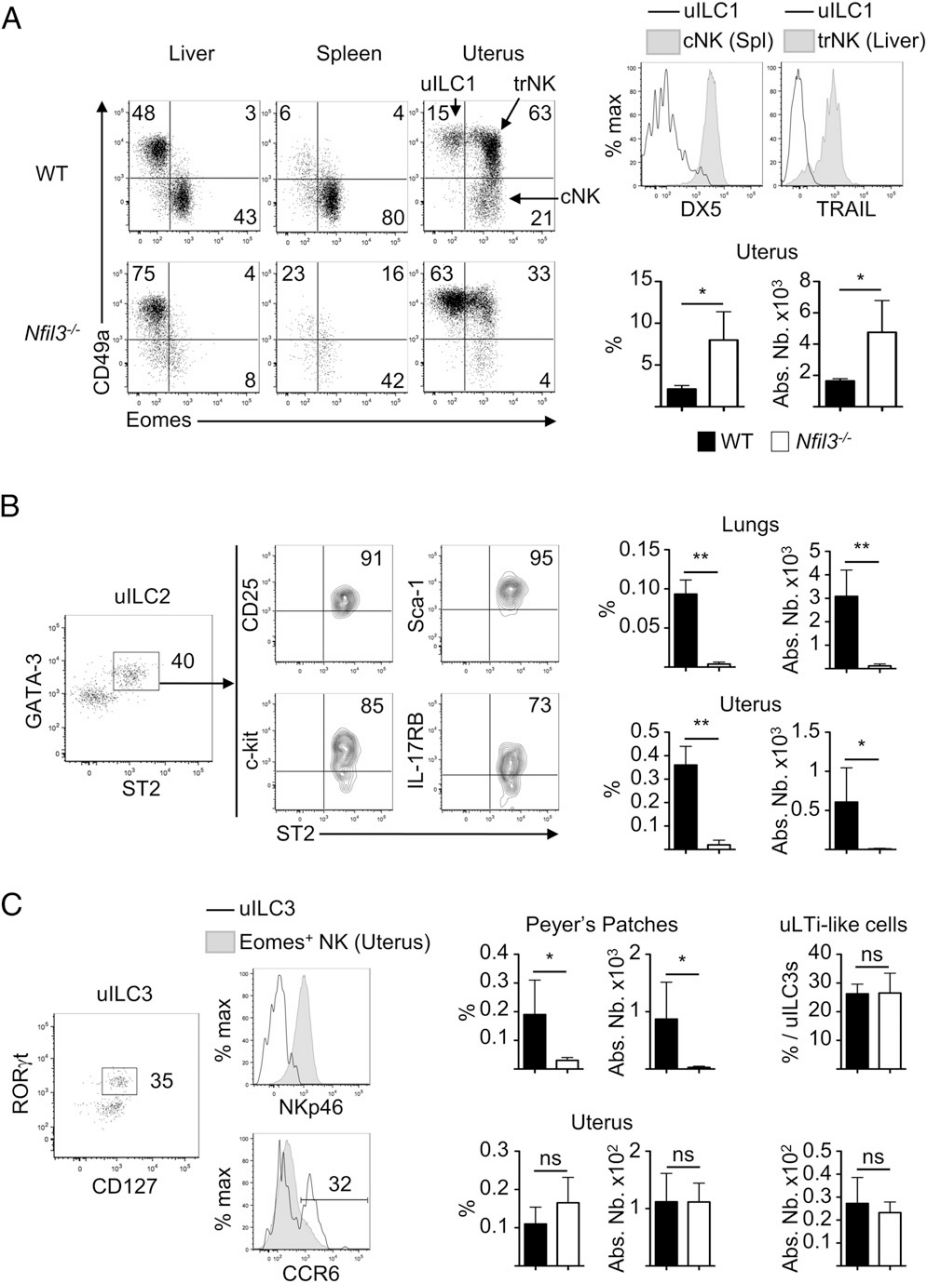
Composition of uILCs in the uterus of virgin WT and *Nfil3^−/−^* mice. (**A**) *Left panel* shows liver, spleen, and uterus CD49a^+^Eomes^−^ ILC1s gated on CD45^+^CD3^−^CD19^−^NK1.1^+^NKp46^+^ cells in virgin WT and *Nfil3^−/−^* mice. Data are representative of four experiments. *Top right panel* shows expression of DX5 and TRAIL on CD45^+^CD3^−^CD19^−^NK1.1^+^NKp46^+^CD90.2^+^T-bet^+^Eomes^−^ WT uILC1s compared with WT splenic cNK cells and WT liver T-bet^+^Eomes^−^ trNK cells, respectively. Data are representative of three experiments. *Bottom right panel* shows percentages among leukocytes and absolute numbers of uILC1s in the uterus of WT (black) and *Nfil3^−/−^* (white) mice (*n* = 4 experiments, mean ± SEM, unpaired *t* test). (**B**) *Left panel* shows GATA-3^hi^ST2^+^ uILC2s gated on CD45^+^CD3^−^CD19^−^CD11b^−^NK1.1^−^NKp46^−^CD90.2^+^ cells from the uterus of virgin mice. Data are representative of 18 experiments. *Middle panel* shows expression of CD25, Sca-1, c-kit, and IL-17RB on uILC2s. Data are representative of five experiments. *Right panels* show percentages among leukocytes and absolute numbers of ILC2s in the lungs (*top panels*) and the uterus (*bottom panels*) of WT (black) and *Nfil3^−/−^* (white) mice (*n* = 2–6 experiments, mean ± SEM, unpaired *t* test). (**C**) *Far left panel* shows RORγt^+^CD127^+^ uILC3s gated on CD45^+^CD3^−^CD19^−^CD11b^−^NK1.1^−^CD90.2^+^ cells. Data are representative of 16 experiments. *Middle left panels* show expression of NKp46 (*n* = 16 experiments) and CCR6 (*n* = 5 experiments) on uILC3s compared with uterine Eomes^+^ NK cells. *Middle right panels* show percentages among leukocytes and absolute numbers of uILC3s in the PP (*top panels*) and the uterus (*bottom panels*) of WT (black) and *Nfil3^−/−^* (white) mice (*n* = 2 experiments, mean ± SEM, unpaired *t* test). *Far right panels* show percentages among leukocytes (*top panels*) and absolute numbers (*bottom panels*) of uLTi-like cells in the uterus of WT (black) and *Nfil3^−/−^* (white) mice (*n* = 2 experiments, mean ± SEM, unpaired *t* test). *p ≤ 0.05, **p < 0.01.

Similarly to the human uterus, the wall of the virgin mouse uterus comprises three layers: the endometrium lining the lumen, the Myo in the middle, and the outermost perimetrium (Supplemental Fig. 1). Besides subsets of uNK cells, which belong to the ILC1 family, nothing is known about composition, development, and function of other uILCs. Two classes of CD3^−^NKp46^+^NK1.1^+^T-bet^+^ ILC1s are known to be present in the virgin mouse uterus: CD49a^+^ trNK-like cells and CD49a^−^DX5^+^ cNK-like cells (19). As in the liver, development of uterine cNK-like, but not uterine trNK-like, cells depends on Nfil3 (19). Similar to splenic cNK cells, uterine cNK-like cells express Eomes and are hence termed uterine cNK cells (Fig. 2A). Analysis of Eomes expression in the uterine CD49a^+^ trNK-like subset revealed two different populations. In contrast with liver trNK cells, the largest subset surprisingly expressed Eomes, named Eomes^+^ uterine trNK cells (Fig. 2A). Uterine trNK cells are therefore different from Eomes^−^ liver trNK cells but similar to Eomes^+^ thymic NK cells and salivary gland NK cells (20, 21). The smallest subset of CD49a^+^ trNK-like cells did not express Eomes and are hereafter defined as uILC1s (CD90.2^+^T-bet^+^Eomes^−^) in the virgin uterine mucosa (Fig. 2A). uILC1s did not express RORγt, whereas GATA-3 was expressed at an intermediate level (data not shown). Therefore, uterine trNK-like NK cells resemble liver T-bet^+^Eomes^−^ trNK cells marked by the expression of CD49a and lacking DX5 expression (Fig. 2A) (4, 30). Once again, we found a striking difference compared with liver T-bet^+^Eomes^−^ trNK cells (4), in that uterine T-bet^+^Eomes^-^ ILC1s do not express TRAIL (Fig. 2A). Furthermore, uILC1s did not express CD127, similar to IEL ILC1s (Supplemental Fig. 2A) (18). In *Nfil3^−/−^* mice, cNK cells were severely reduced in both liver and spleen, whereas uILC1s were present and increased in frequency and in absolute number, becoming the most abundant subset within the CD49a^+^ cells (Fig. 2A). Altogether, these results highlight the complexity of the ILC1s in the uterus, including at least three subsets: uILC1s, trNK cells, and cNK cells.

### Development of mouse uILC2s depends on Nfil3

uILC2s were present among the CD45^+^CD3^−^CD19^−^CD11b^−^NK1.1^−^NKp46^-^CD90.2^+^ cells in the virgin uterus (Fig. 2B) and expressed GATA-3 as well as surface markers ST2, CD25, c-Kit, Sca-1, IL-17RB, and, at intermediate levels, CD127 (Fig. 2B, Supplemental Fig. 2A). uILC2s also expressed ICOS, which is, however, also found on other CD90.2^+^ cells expressing low GATA-3 (Supplemental Fig. 2B). The combined expression of ST2 and GATA-3 may be the best strategy to define uILC2s. In *Nfil3^−/−^* mice, uILC2s were severely reduced as previously described in the lungs (Fig. 2B).

### Development of mouse uILC3s does not depend on Nfil3

uILC3s were found in the virgin uterus and are identified as CD45^+^CD3^−^CD19^−^CD11b^−^NK1.1^−^CD90.2^+^RORγt^+^ cells (Fig. 2C). The presence of uILCs in *Rag2^−/−^* mice demonstrates that their presence is not the result of T cell contamination (Supplemental Fig. 3). uILC3s expressed higher level of CD127 compared with other uILCs (Supplemental Fig. 2A). A more in-depth analysis of uILC3s revealed the presence of CCR6^+^ LTi-like cells and CCR6^−^NKp46^−^ ILC3s in the virgin uterus, but no CCR6^−^NKp46^+^ ILC3s (Fig. 2C). In *Nfil3^−/−^* mice, as reported previously, the PP showed marked reduction in number per mouse, in total cell number per PP, and in size (Supplemental Fig. 4). We also found no ILC3s in the PP, confirming previous studies showing that Nfil3 is required for ILC3s and for the generation of the PP (Fig. 2C). Surprisingly, however, both uILC3s and uterine LTi (uLTi)-like cells were present and in normal numbers in the *Nfil3^−/−^* virgin uterus (Fig. 2C).

### Mouse uILC2s and uILC3s are absent from the decidua

**FIGURE 3.**
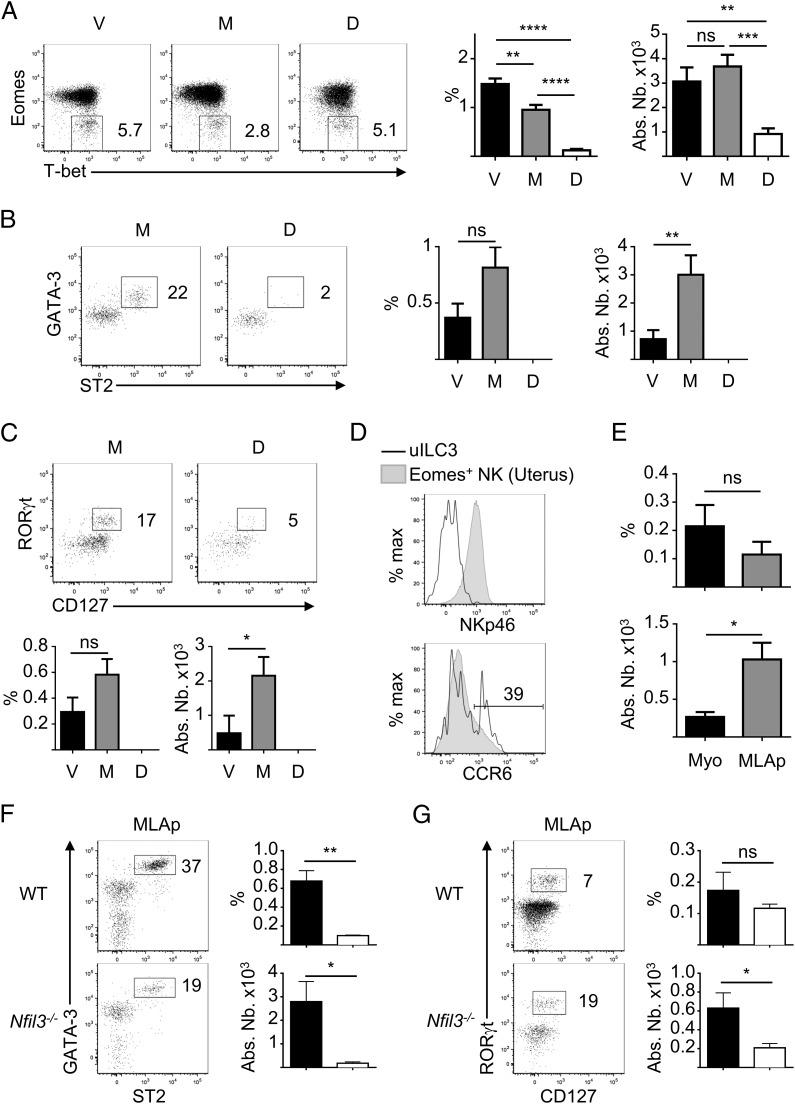
Distribution of ILCs in the layers of the uterine wall of pregnant mice. (**A**) *Left panel* shows percentages of T-bet^+^Eomes^−^ uILC1s in the uterine wall of virgin mice (V), compared with percentages in uterine layers of pregnant mice at midgestation, dissected out in Myo and MLAp together (M) and decidua (D). Data are representative of six experiments. *Right panels* show percentages among leukocytes (*left*) and absolute numbers (*right*) of uILC1s per mouse (*n* = 6 experiments, mean ± SEM, unpaired *t* test). (**B**) Representative dot plots (*left panel*) show percentages of GATA-3^hi^ST2^+^ uILC2s in uterine layers of pregnant mice at midgestation, dissected as in (A). Data from virgin mice are shown in Fig. 2B. Data are representative of eight experiments. *Right panels* show percentages among leukocytes (*left*) and absolute numbers (*right*) of uILC2s per mouse (*n* = 8 experiments, mean ± SEM, unpaired *t* test). (**C**) *Top panels* show percentages of RORγt^+^CD127^+^ uILC3s in uterine layers of pregnant mice at midgestation, dissected as in (A). Data from virgin mice are shown in Fig. 2C. Data are representative of seven experiments. *Bottom panels* shows percentages among leukocytes (*left*) and absolute numbers (*right*) of uILC3s per mouse (*n* = 7 experiments, mean ± SEM, unpaired *t* test). (**D**) Histograms show expression of NKp46 (*n* = 12 experiments) and CCR6 (*n* = 3 experiments) on uILC3s from the Myo and the MLAp dissected together and compared with conventional uNK cells. (**E**) Percentages among leukocytes (*top panel*) and absolute numbers (*bottom panel*) of CCR6^+^ uLTi-like cells per mouse in the isolated Myo and the isolated MLAp dissected apart (*n* = 2 experiments, mean ± SEM, paired *t* test). (**F** and **G**) Representative dot plots (*left panel*), percentages among leukocytes (*top right*) and absolute numbers (*bottom right*) of GATA-3^hi^ST2^+^ uILC2s (F) and RORγt^+^CD127^+^ uILC3s (G) in the MLAp at midgestation (gd9.5 to gd11.5) in WT (black) and *Nfil3^−/−^* (white) mice (*n* = 3 experiments, mean ± SEM, unpaired *t* test). *p ≤ 0.05, **p < 0.01, ***p < 0.001, ****p < 0.0001.

**FIGURE 4.**
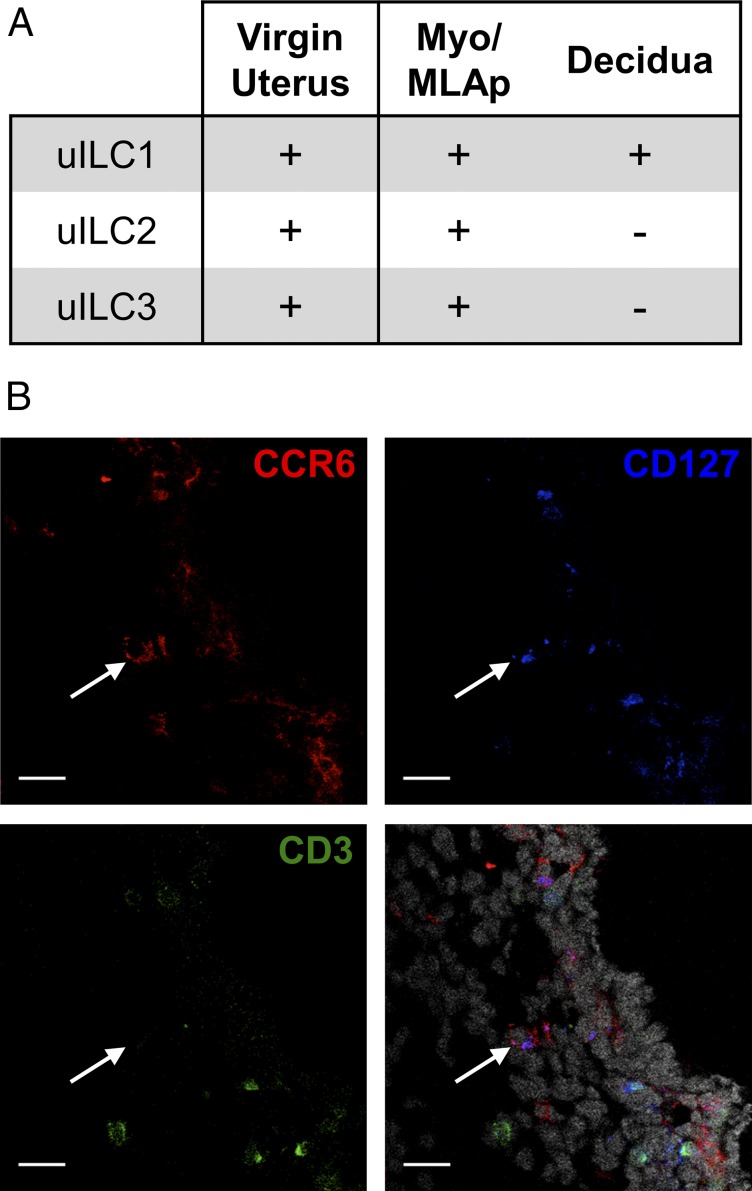
Distribution of uILCs in the uterus of virgin and pregnant mice at midgestation. (**A**) The Myo and the MLAp contain uILC1, uILC2, and uILC3 populations. Only uILC1s are identified in the decidua. All three populations are also identified in the mesometrial tissue connecting adjacent implantation sites. (**B**) Immunofluorescence staining of CCR6 (red), CD127 (blue), and CD3 (green) in mouse Myo at gd9.5. The presence of a CCR6^+^CD127^+^CD3^−^ uLTi-like ILC3 is indicated by an arrow in each fluorescence channel. Scale bar, 20 μm.

uILC1s could be found at midgestation in both Myo/MLAps and decidua, albeit at lower frequencies compared with the virgin uterus (Figs. 3A, 4A, Supplemental Fig. 1). uILC2s and uILC3s were found in the Myo/MLAp, but not in the decidua (Fig. 3B, 3C); thus, uILC2s and uILC3s do not interact directly with the trophoblast and instead sit deeper in the uterine wall (Fig. 4, Supplemental Fig. 1). The frequency of uILCs at midgestation was similar to that of the virgin uterus, suggesting that they did not preferentially expand among leukocytes. However, we noticed a trend toward a higher percentage of uILC2s (*p* = 0.06) and uILC3s (*p* = 0.10) in the Myo/MLAp compared with the virgin uterus (Fig. 3B, 3C). To assess the distribution of uLTi-like cells, we dissected the MLAp and the Myo apart and they were found to contain similar frequencies of uLTi-like cells. Because of a high concentration of lymphocytes in the MLAp, however, the absolute number of uLTi-like cells was significantly higher in the MLAp compared with the Myo (Fig. 3E). We then assessed the impact of pregnancy on the development of uILC2s and uILC3s in the MLAp of *Nfil3^−/−^* mice. uILC2s were present but at very low percentages and numbers showing that pregnancy cannot rescue their development (Fig. 3F). As in virgin mice, uILC3s are detectable at a similar percentage to wild-type (WT) mice, but numbers are significantly lower, reflecting the reduced numbers of leukocytes in the MLAp of *Nfil3^−/−^* mice (Fig. 3G). Indeed, *Nfil3^−/−^* mice display several defects during pregnancy (S. Boulenouar, J.-M. Doisne, A. Sferruzzi-Perri, L.M. Gaynor, J. Kieckbusch, E. Balmas, H.W. Yung, S. Javadzadeh, L. Volmer, D.A. Hawkes, K. Phillips, H.J.M. Brady, A.L. Fowden, G.J. Burton, A. Moffett, and F. Colucci, submitted for publication). Taken together, these data show that uILC1s are present in all layers of the pregnant uterus, whereas uILC2s and uILC3s are confined deeper within the muscle layer (Fig. 4). In addition, during pregnancy in *Nfil3^−/−^* mice, uILC2 development is not rescued and uILC3s decrease in numbers compared with pregnant WT mice.

### Mouse uILC2s constitutively produce IL-5, but not IL-13, and uILC3s constitutively produce IL-17 and IL-22

**FIGURE 5.**
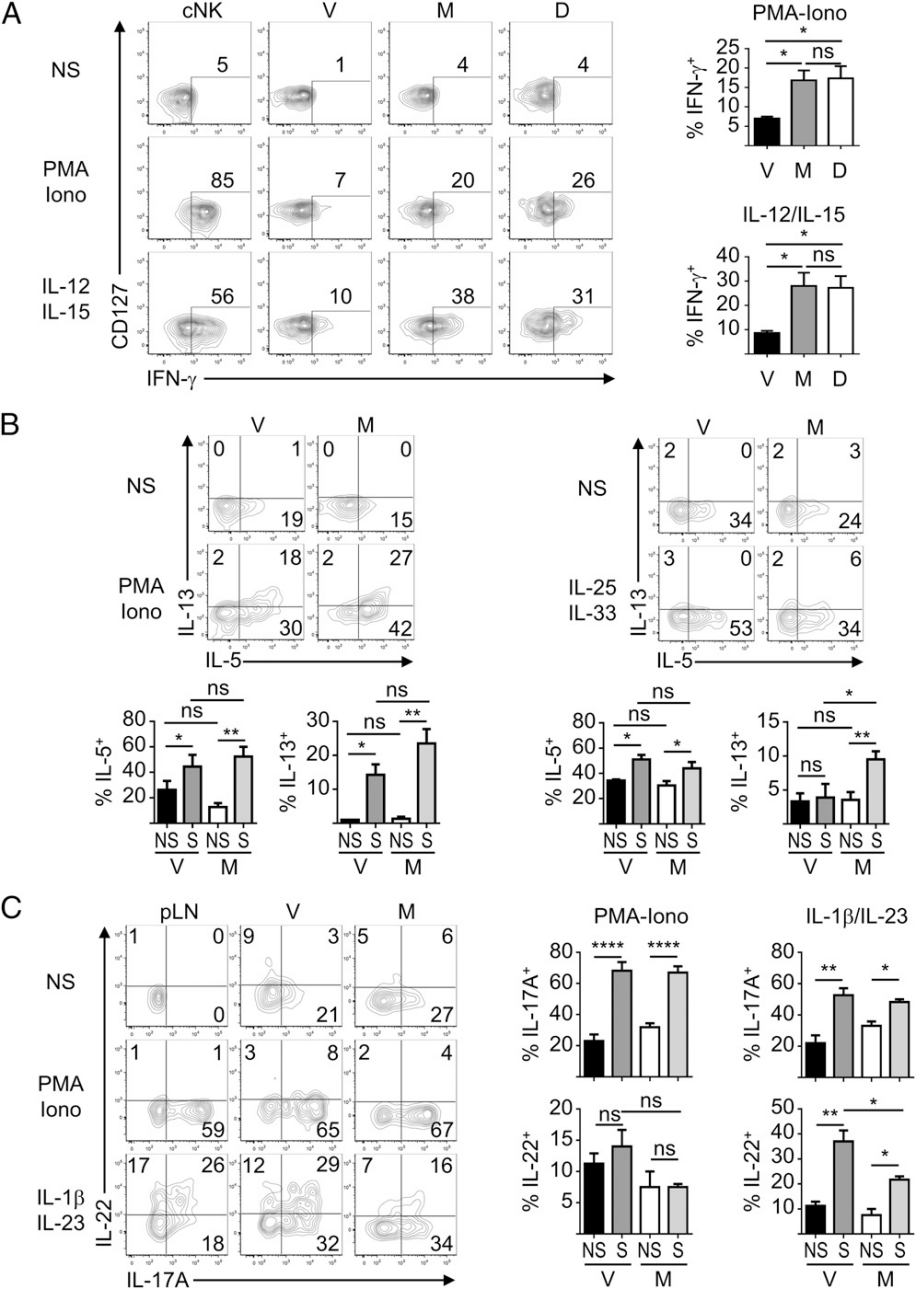
Cytokine production by uILCs. (**A**) Intracellular IFN-γ in cNK cells from the spleen and in uILC1 from V, M, and D was measured upon stimulation ex vivo with either PMA and ionomycin or IL-12 and IL-15. *Left panel* shows data for gated uILC1s. *Right panels* show percentages of IFN-γ^+^ cells in stimulated (S) or unstimulated (NS) uILC1s (*n* = 3–6 experiments, mean ± SEM, unpaired *t* test). (**B**) Intracellular IL-5 and IL-13 in cells from V and M were measured upon stimulation ex vivo with either PMA and ionomycin or IL-25 and IL-33. *Top panels* show data for gated uILC2s. *Bottom panels* show percentages of IL-5^+^ cells and IL-13^+^ cells among S and NS uILC2s (*n* = 2–6 experiments, mean ± SEM, paired *t* test). (**C**) Intracellular IL-17A and IL-22 in cells from peripheral lymph nodes (pLN); V and M were measured upon stimulation ex vivo with either PMA and ionomycin or IL-1β and IL-23. *Left panels* show data for gated uILC3s. *Right panels* show percentages of IL-17A^+^ cells and IL-22^+^ cells among S and NS uILC3s (*n* = 2–6 experiments, mean ± SEM, paired *t* test). *p ≤ 0.05, **p < 0.01, ****p < 0.0001.

uILC1s produced IFN-γ upon short stimulation in vitro with either PMA and ionomycin or with IL-12 and IL-15 (Fig. 5A). Compared with splenic cNK cells, fewer uILC1s produced IFN-γ, although the frequency of IFN-γ^+^ upon stimulation increased at midgestation (Fig. 5A).

uILC2s produced IL-5 constitutively in the virgin uterus and more so by midgestation, whereas only a few uILC2s made IL-13 upon stimulation (Fig. 5B). uILC2s appeared to be the only source of IL-5 either constitutively produced or induced after short activation in the uterus, because we did not detect any other IL-5^+^ cells. IL-13 was not induced by IL-25 and IL-33 in the virgin uterus, suggesting that uILC2s are possibly more responsive to these cytokines during pregnancy because IL-13 production could be induced in the Myo/MLAp. Alternatively, uILC2s may need other cytokines, such as IL-2 or IL-7, to be fully activated. IL-13 was mainly expressed by IL-5–producing cells (Fig. 5B), and as for IL-5, uILC2s were the only source of IL-13 in the uterus after a short activation period.

uILC3s constitutively produced IL-17A and IL-22, unlike ILC3s from peripheral lymph nodes (Fig. 5C). Production of IL-17A and IL-22 was enhanced upon stimulation, especially for IL-17A (Fig. 5C). Intriguingly, stimulation with PMA and ionomycin did not enhance IL-22 production, whereas stimulation with IL-1β and IL-23 did (Fig. 5C). Uterine immune cells other than uILC3s, for example, RORγt^+^CD3^+^ iNKT17 cells (31), were also able to secrete IL-17 and IL-22 quickly in both virgin and pregnant mice (data not shown).

## Discussion

uNK cells are the likely key immune players at the maternal–fetal interface and may also play roles in endometrial homeostasis (22). The potential role of other ILCs in the uterus is unknown. We have shown that only uILC3s are present in human endometrium and decidua, whereas all three groups of murine ILCs are present in the virgin uterus, as well as in the Myo and MLAps of dams at midgestation. We also report in this article the discovery that development of mouse uILC3s is not Nfil3 dependent, in contrast with development of ILC3s present in other tissues, as previously described. Functionally, we find that both uILC2s and uILC3s constitutively produce IL-5 and IL-17/IL-22, respectively.

### Nfil3 and uILC development

ILC development depends on Nfil3 (15–17), and we show in this article that uILC2s are indeed dependent on Nfil3 similar to lung, fat, and intestine ILC2s. These results suggest that ILC2 development may be homogeneous and not tissue-specific. However, during pregnancy, very few uILC2s can be detected. These residual uILC2s may expand by homeostatic proliferation, filling the space left by the severe reduction in uNK cells in *Nfil3^−/−^* mice. In contrast, both uILC1s and uILC3s are present in *Nfil3^−/−^* mice with an increase in the Eomes^−^ uILC1s, a population that may include developmentally arrested CD49a^+^Eomes^+^ trNK cells. Indeed, Nfil3 is crucial for Eomes expression (32). The developmental relationship among uILC1s, trNK, and cNK is unknown. Some ILC1-like cells directly derive from NCR^+^ ILC3s (8–10), but the lack of NCR^+^ ILC3s should exclude this possibility, although fate-mapping experiments will be needed to test this (13). Interestingly, we find both NCR^−^ and LTi-like uILC3s in *Nfil3^−/−^* mice, whereas ILC3s are absent in their PP. This could reflect compensatory mechanisms in the uterine environment or a different developmental pathway. For example, Eomes expression in the uterus dominates over T-bet expression (33). During pregnancy, uILC3s in *Nfil3^−/−^* mice do not expand to the same extent as in WT mice, and in the future it will be of interest to test whether this effect is intrinsic or caused by the changes in the cellular environment, notably the severe reduction in uNK cells. Nfil3 is not required for extramedullary ILC1 development (21), and other extramedullary ILC subsets may follow similar developmental pathways, independent of Nfil3, whereas ILCs originating from the bone marrow may be strictly dependent on Nfil3.

### uILC1

A recent article described two putative CD127^−^ ILC1 subsets in human decidua: CD56^+^T-bet^+^Eomes^+^ cells and CD56^−^T-bet^+^Eomes^−^ cells, which resemble the mouse uILC1s described in this article (23). The identity of these cells is still undefined and they might also be immature NK cells or ILC3-derived ILC1s. We did not find CD127^+^ uILC1s in human endometrium or decidua. However, we cannot exclude the presence of uILC1s in the basal layer of the mucosa or in the Myo because of difficulties in sampling these sites. The uILC1s detected in WT mice may be immature NK cells, but this is unlikely because they are CD127^−^, produce IFN-γ, and are found in *Nfil3^−/−^* mice. IFN-γ is a key factor for arterial remodeling during pregnancy in mice (22). Although uNK cells produce the most IFN-γ in the uterus of pregnant mice, uILC1s may also contribute to IFN-γ production. IFN-γ produced by Eomes^−^ ILC1s upon cytokine stimulation increases during pregnancy, reflecting a change in their activation status and/or a more favorable microenvironment that induces activation. *Tbet^−/−^* mice, however, show normal spiral artery remodeling (33). The role and function of Eomes^+^ trNK cells is currently under investigation (S. Boulenouar et al., submitted for publication).

### uILC2

ILC2s were found deep in the uterine wall and not in human or murine decidua, nor in human endometrium. Whether they are present in human Myo, therefore, is still unknown, but there is no equivalent structure to the MLAp in humans. In mice, uILC2s constitutively produce IL-5, confirming findings using an IL-5 reporter mouse model, where ILC2s were shown to control eosinophil homeostasis through an IL-5–dependent mechanism (34). In mice, eosinophils may play a role in tissue remodeling of the uterine mucosa (35) (although they are not present in humans), and it will be interesting to study the relationship between uILC2s and uterine eosinophils.

uILC2s respond to IL-25 and IL-33, both previously found in the uterine mucosa during pregnancy. Lung ILC2s produce amphiregulin and GM-CSF, and these cytokines, if produced by uILC2s, may also play important roles in uterine tissue remodeling and trophoblast migration. IL-5^−/−^ and IL-13^−/−^ mice, however, do not show any obvious phenotypes in pregnancy (35, 36).

### uILC3

NCR^+^ uILC3s and LTi-like uILC3s are present in both human endometrium and decidua with similar frequencies and with similar phenotype, suggesting a stable uILC composition despite the changes in the mucosa during decidualization. Similar to our study, uILC3s were found in human decidua (23). Human uILC3s are likely to include the IL-22–producing NK cells we described previously and referred to then as stage 3 NK cells (37). Indeed, these cells are CD9^−^c-kit^+^CD127^+^, whereas CD56 and NKp44 are expressed heterogeneously. It will be interesting to dissect the developmental relationship among stage 3 NK cells, NCR^+^ uILC3s, and LTi-like uILC3s. A recent article shows that AHR inhibition in tonsil ILC3s induces NK cell differentiation, highlighting the close relationship between ILC3s and NK cells (38). Along these lines, differentiation of IFN-γ–producing uNK cells was abnormal in *AhR^−/−^* mice (39).

In mice, uILC3s comprise uLTi-like cells and NCR^−^ cells. The lack of NCR^+^ ILC3s is surprising because it is thought that they originate from NCR^−^ ILC3s. Mouse uLTi-like ILC3s are distributed throughout the uterine wall, including the MLAp. The location of uLTi-like ILC3s, in proximity to uILC1s and uNK cells, suggests that uLTi and uILC1s, besides uNK cells, may also contribute to changes in the uterine vasculature during pregnancy particularly as the main uterine vessels enter the uterine wall through the MLAps in mice (Supplemental Fig. 1). Gut LTi cells play a role in lymphoid organogenesis, and thus uLTi-like cells could organize formation of MLAp in mice. However, LTα^−/−^ and LTβR^−/−^ pregnant mice do develop MLAp, although both decidual development and cellularity of the MLAp are delayed in both these mutant mice (28). Furthermore, no obvious uNK cell defects were found in these mice (28), although a recent study shows that ILC3s are able to support NK cell development through LT-mediated stromal microenvironment (40). Other soluble factors, such as IL-22, may play a role in the development and maintenance of the MLAp. IL-22 has both proinflammatory and tissue-protective roles in the gut and the skin. Moreover, along with Th17 cells, uILC3s may promote survival, proliferation, and invasion of human trophoblast during the first trimester of pregnancy (41, 42). uILC3s typically respond to IL-1β and IL-23, known to be produced by macrophages and DCs. IL-1β can be produced by human uterine macrophages and fetal trophoblast (43, 44), whereas IL-23 produced by stromal cells is detected in pregnant mice (44, 45).

We report in this article the presence of ILC3s in the human uterus and the presence of all three groups of ILCs in the mouse uterus where the development of uILC1s and uILC3s is Nfil3 independent. The differences in composition between human and mouse uILCs may reflect the dramatic changes the mucosa undergoes in the human menstrual cycle with cyclical degeneration and renewal. In addition, we have only sampled the early stages of pregnancy in humans and not later in gestation, and have not been able to analyze immune cells in the basal layer of the mucosa or Myo. In the future, it will be interesting to study the developmental pathways of uILCs, their interactions with uterine leukocytes, including NK cells and macrophages, as well as with the uterine vasculature and stroma, and ultimately their role in normal and pathological pregnancy.

## Supplementary Material

Data Supplement
