# Supplementary material for: Composition, Development, and Function of Uterine Innate Lymphoid Cells
Source: J Immunol. 2015 Sep 14;195(8):3937–45. doi: 10.4049/jimmunol.1500689 (PMC4592103; doi:10.4049/jimmunol.1500689)
Supplement: Data Supplement [file JI_1500689.zip › JI_1500689_Supplemental_Figures_1.pdf]

Supplementary Figure 1

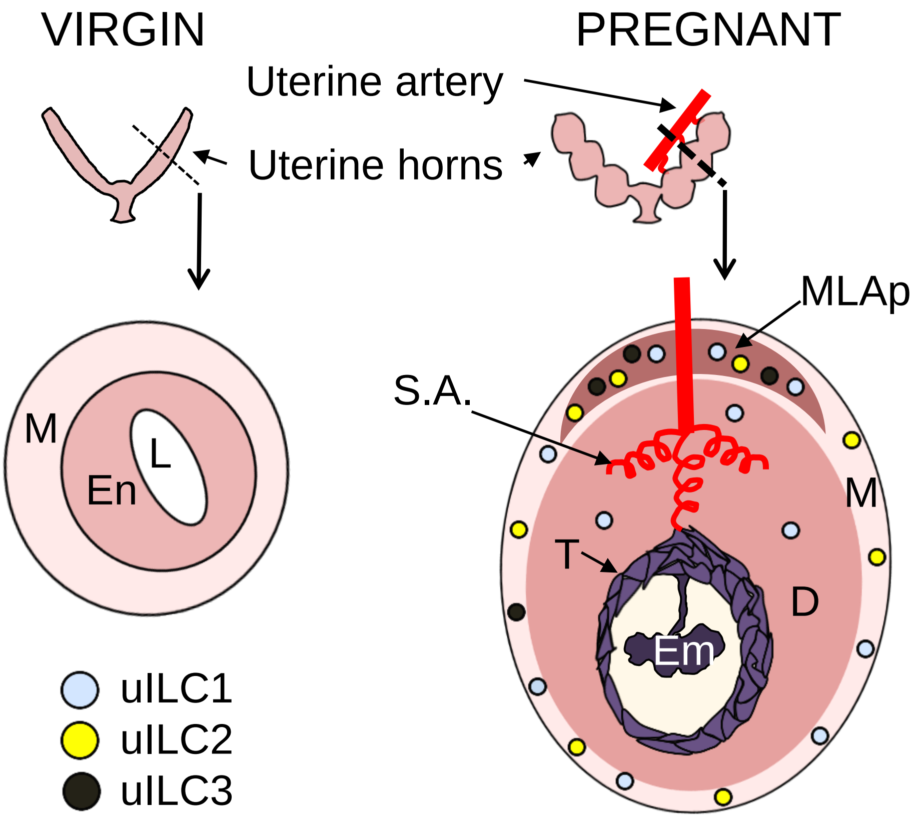

Figure S1. Anatomical distribution of uILCs. The diagram depicts a section cut perpendicularly to the long axis of the uterine horn from a virgin (left) and from a pregnant uterus at midgestation (right). Spiral arteries (S.A.) in the decidua (D) undergo remodelling at midgestation, which transforms them to vessels of high conductance to adequately supply the feto-placental unit with maternal blood. Spiral arteries originate from the uterine artery, which traverses the MLAp before entering the decidua. The myometrium and the MLAp contain uILC1, uILC2 and uILC3 populations. Only uILC1s are identified in the decidua. All 3 populations are also identified in the mesometrial tissue connecting adjacent implantation sites. M: Myometrium; L: Lumen; En: Endometrium; T: Trophoblast; Em: Embryo.

Supplementary Figure 2

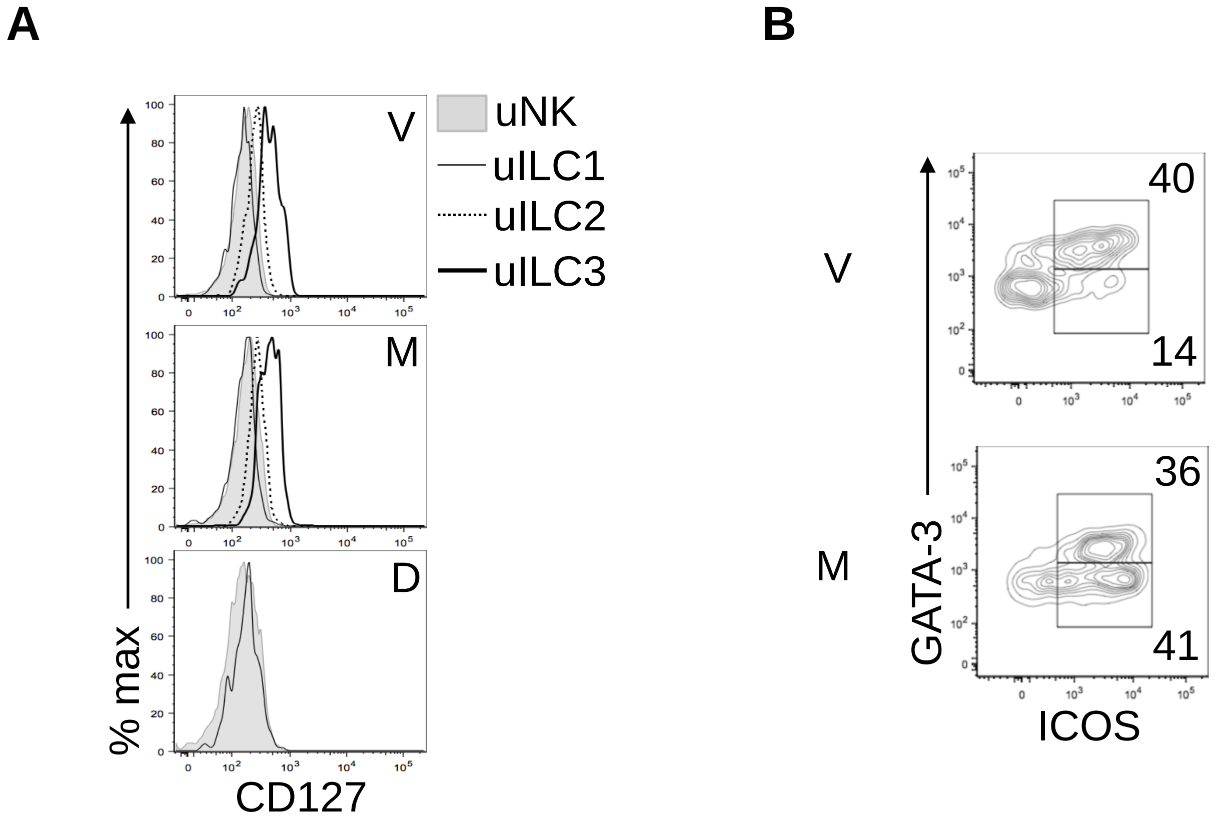

Figure S2. CD127 expression on uILCs and ICOS expression on uILC2s. A. Histograms show CD127 expression levels on uILCs compared to uterine cNK cells in V, M and D. Data are from at least 7 experiments. **B.** ICOS expression among GATA-3^{hi} (uILC2s) and GATA-3^{lo} cells was determined in the virgin uterus and the Myo/MLAp. Dot plots are gated on live CD45^{+}CD3^{-}CD19^{-}CD11b^{-}NK1.1^{-}NKp46^{-}CD90.2^{+} cells and are representative of 3 experiments.

Supplementary Figure 3

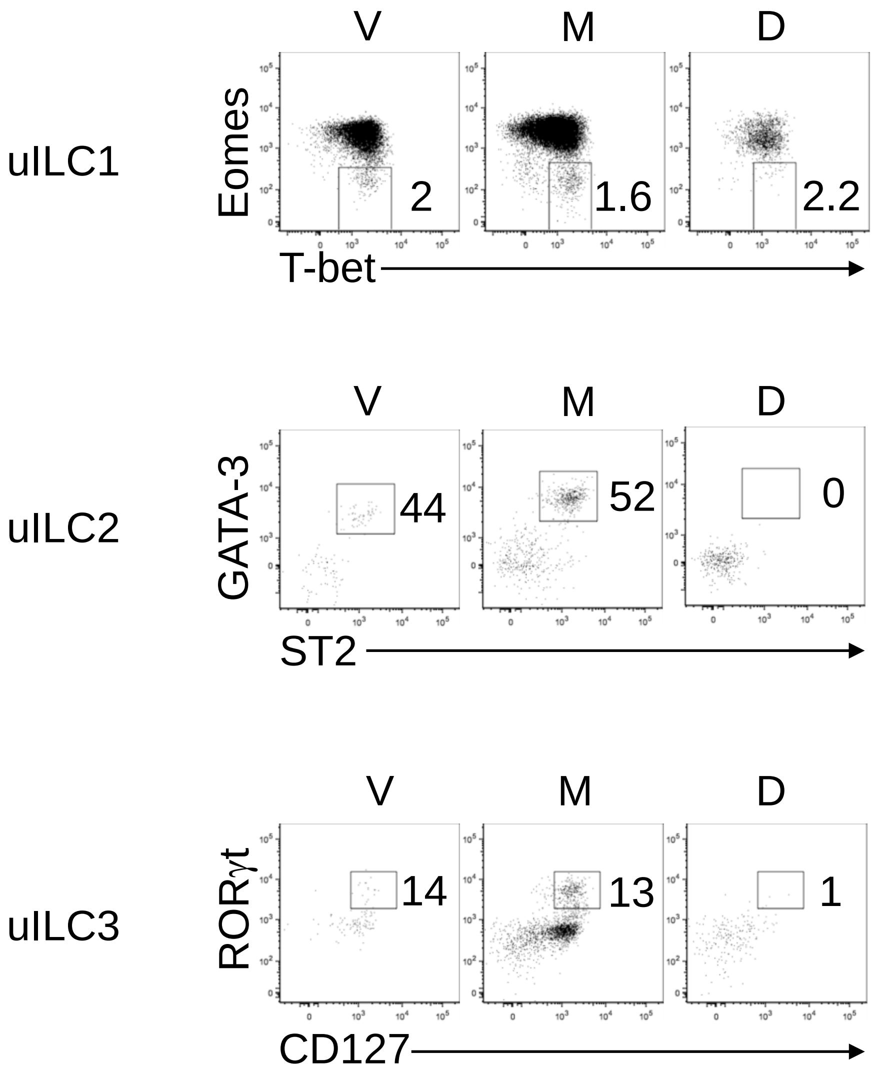

Figure S3. uILCs analysis in the uterus of *Rag2*$^{-/-}$ mice. uILC1s (top panel), uILC2s (middle panel) and uILC3s (bottom panel) are present in the uterus of virgin *Rag2*$^{-/-}$ mice. Data are from 2 experiments.

Supplementary Figure 4

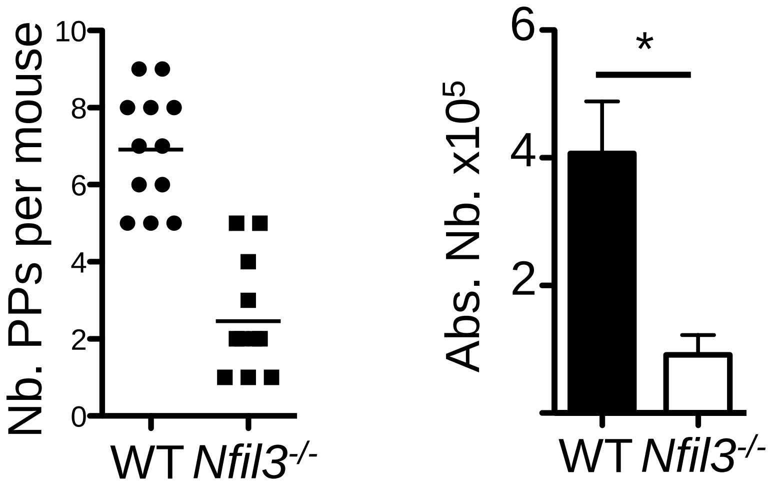

Figure S4. Peyer's patches analysis in *Nfil3*$^{-/-}$ mice. The left graph shows the number of Peyer's patches collected per small intestine. Each dot represents an individual mouse. The bar corresponds to the mean. Data collected from 2 independent experiments. The right graph shows the absolute leukocyte numbers per Peyer's patch (n = 2 experiments, mean ± SEM, unpaired *t*-test).
